# Student self-esteem in music education contexts: a systematic literature review

**DOI:** 10.3389/fpsyg.2025.1515305

**Published:** 2025-10-13

**Authors:** Xin Jiang, Yanli Tong

**Affiliations:** ^1^Department of Music and Education, Chuzhou University, Chuzhou City, Anhui, China; ^2^Faculty of Language and Culture, Ningde Normal University, Ningde, China

**Keywords:** self-esteem, student, music education, PRISMA, systematic literature review

## Abstract

**Introduction:**

This systematic literature review (1970–2023) examines how music education relates to students’ self-esteem. Although interest has grown since 1970, evidence remains dispersed across designs and populations.

**Methods:**

Following the PEO (population, exposure, outcome) framework and PRISMA guidelines, we systematically searched EBSCO, Scopus, and Web of Science. Of 1,332 records identified, we screened empirical studies of students engaged in formal or informal music learning that reported self-esteem outcomes. We extracted trends, methods, measurement tools, and results, and appraised methodological quality.

**Results:**

Twenty-two studies met inclusion criteria (18 quantitative; 4 mixed-methods), with a predominance of cross-sectional designs. Quantitative studies showed stronger methodological quality (mean, x̅ = 0.79), whereas qualitative components of mixed-methods studies were lower (x̅ = 0.43). The Rosenberg Self-Esteem Scale was the most widely used instrument. Across studies, self-esteem in music education was associated with demographic factors (e.g., gender, age) and psychological factors (e.g., self-efficacy). Overall, music learning was linked to enhanced self-esteem among children and adolescents, with notable benefits for specific student groups.

**Discussion:**

Current evidence indicates a positive relationship between music education and students’ self-esteem; however, generalizability is limited by the dominance of cross-sectional designs and uneven methodological quality. Future research should employ broader designs (e.g., longitudinal and experimental), include more diverse populations, and use consistent, validated measures to strengthen causal inference and applicability.

## 1 Introduction

Self-esteem is a core construct in social and educational psychology, commonly defined as an individual’s overall evaluation of self-worth—a sense of self-acceptance and value rather than superiority or perfection (Mruk, 2013). Research has shown that self-esteem is a key predictor of mental health and academic achievement (Kärchner et al., 2021), closely related to positive affect and self-efficacy (Zuffianò et al., 2023), while low self-esteem is often associated with depression, anxiety, and academic burnout (Zapata-Lamana et al., 2021). In educational contexts, students’ self-esteem influences motivation and classroom engagement, underscoring its developmental significance (Acosta-Gonzaga, 2023). Theoretically, Shavelson et al. (1976) multidimensional, hierarchical model posits that self-esteem derives from self-concepts across specific domains, which together form global self-esteem. Global self-esteem refers to individuals’ overall evaluation of self-worth, a relatively stable and broad psychological trait predictive of a wide range of behaviors and adaptive outcomes (Hattie, 2014; Turner and Onorato, 1999). Measurement tools reflect these orientations: for instance, the Rosenberg Self-Esteem Scale assesses general self-worth (Monteiro et al., 2022), whereas the Coopersmith Self-Esteem Inventory incorporates academic, social, and familial domains, reflecting a multidimensional perspective (Coopersmith, 1981; Ozturk and Acikgoz, 2022).

Recent scholarship has refined multidimensional models of self-esteem and highlighted the importance of domain-specific approaches in education (Marsh et al., 2018; Rentzsch et al., 2021). In the context of music education, beyond global self-esteem, music self-esteem refers to individuals’ evaluations of their musical skills, performance, and learning outcomes, reflecting both subjective value and perceived competence (Culp, 2016). Empirical evidence further links musical self-esteem to social competence, music preference, and well-being (Fancourt et al., 2022; Saragih and Rahmatulloh, 2024).

Musical self-esteem is increasingly conceptualized as a multidimensional construct that integrates self-perceptions of ability, social recognition, and personal aspirations—dimensions closely tied to skill development, motivation, belonging, and sustained engagement (Sandgren, 2019; Oliveira et al., 2021). This perspective not only clarifies the distinction between global and domain-specific self-esteem but also provides a stronger theoretical basis for examining how music education contributes to students’ self-worth and psychological development.

Music instruction refers to structured courses and learning activities aimed at developing students’ musical skills and knowledge (McPherson et al., 2012). By contrast, music education encompasses a broader scope, including not only musical skill acquisition but also cultural understanding, emotional expression, social interaction, and holistic development (Reimer, 2022; Váradi, 2022). Music instruction can therefore be regarded as a core component within the wider framework of music education.

Research on self-esteem in the music context has recently attracted increasing interest. It has been demonstrated that music is a valid method for enhancing self-confidence, thus fostering feelings of happiness and contentment (Creech et al., 2014). Additionally, music has been shown to affect individuals’ self-esteem, alleviate burnout, and further enhance self-confidence (Sharma and Jagdev, 2012). In the context of music education, studies have shown that among college-level music students, self-esteem is significantly and negatively correlated with music performance anxiety, indicating that students with higher self-esteem tend to experience lower levels of performance anxiety (Otacioglu, 2016). Both low self-esteem and music performance anxiety are believed to lead to depression in musicians (Sickert et al., 2022). Moreover, Rickard et al. (2013) found that including more music classes in schools had a positive effect on children’s social skills and self-esteem.

Music education holds distinctive advantages over other art forms in fostering self-esteem. Even passive music listening, through melody, rhythm, and emotional content, can trigger emotional regulation and positive affect, thereby enhancing both explicit and implicit self-esteem (Elvers et al., 2018). Beyond listening, active engagement in music learning promotes self-efficacy and self-esteem through skill development and mastery experiences (Jiang, 2024). Moreover, collaborative practices such as choir and ensemble playing strengthen social self-esteem and a sense of belonging (Verneert et al., 2021). Collectively, these mechanisms underscore the unique potential of music education—distinct from other art forms—in promoting self-esteem.

Lawendowski and Bieleninik (2017) were the only researchers to review studies on self-esteem in the field of music therapy prior to 2017. They examined the psychological processes associated with specific musical practices that influence how individuals perceive themselves and their self-esteem, which, in turn, can affect their overall sense of personal identity. However, no systematic synthesis has yet examined the relationship between global and music-specific self-esteem in the context of music education. This gap limits comprehensive understanding and constrains the generalizability and applicability of existing findings across diverse contexts.

This study synthesizes research conducted between 1970 and 2023, systematically evaluating the methodological rigor and key findings to inform future directions. In addition to summarizing the relationship between music education and self-esteem, the review offers empirical insights that contribute to cross-cultural educational practice and policy development, underscoring its theoretical and practical significance. By identifying existing limitations, the review emphasizes the need for more robust research designs and a greater diversity of participant samples to foster a comprehensive understanding of how music education influences self-esteem.

This review is guided by the following research questions:

**RQ1**: What are the key research trends in music education and self-esteem from 1970 to 2023?

**RQ2**: What methodologies, research designs, and participant populations have been utilized in this field?

**RQ3**: What self-esteem indicators and measurement tools are applied in music education research?

**RQ4**: What is the methodological quality of the studies included, and what strengths and limitations can be identified?

**RQ5**: What are the key findings regarding students’ self-esteem in music education across diverse contexts?

By addressing these questions, this review highlights the significance of the topic and identifies critical areas for future research, particularly the need for more diverse study designs and participant populations.

## 2 Method

This systematic review followed the PRISMA guidelines (Moher et al., 2010) and was structured according to the Population, Exposure, and Outcome (PEO) framework (Caparros-Gonzalez et al., 2021). The review process comprised four main steps: (a) conducting a systematic search and rigorous screening of relevant literature; (b) examining the influence of demographic variables (e.g., gender, age, cultural background) and methodological features (e.g., research design, measurement tools, sample size) on outcomes; (c) assessing the methodological quality of the selected studies using the Kmet et al. (2004) criteria; and (d) situating findings within multidimensional self-esteem theory to explore the connections between global and music-specific self-esteem. Additionally, (e) key gaps in the literature were identified, including the lack of cross-cultural research, limited validation of measurement tools, and the scarcity of intervention and longitudinal designs, highlighting priorities for future investigation. The review primarily focused on observational studies but also included studies with intervention-like characteristics when relevant. The term “exposure” was broadly defined to encompass any structured music-related activity or curriculum, including music therapy sessions with pedagogical aims.

The outcomes of the review were twofold: (a) publication metrics of the included studies, such as their number, bibliometric performance, and quality; and (b) the psychological and educational effects of music instruction on students. By integrating both domains, the review emphasizes the academic contributions of music instruction research while underlining its positive impact on students’ self-esteem. Despite the diverse research designs and typologies employed, such as quantitative surveys and qualitative case studies, each study type offered valuable insights. Quantitative studies provided broad statistical findings, while qualitative studies offered in-depth contextual understanding. The combination of both methodologies strengthened the review by incorporating a range of perspectives on the relationship between music education and self-esteem.

Although this review was not pre-registered on PROSPERO, it adhered strictly to the PRISMA guidelines, with transparent reporting of search strategies, inclusion and exclusion criteria, data extraction, and analytic procedures. This adherence ensures the reproducibility and rigor of the review, enhancing its credibility (Page et al., 2021; Cumpston et al., 2019).

### 2.1 Database searches

This review adopted 1970 as the starting point, given the fragmented nature of early research on self-esteem in music education and the use of standardized measures like the Rosenberg Self-Esteem Scale during this period (Schmitt and Allik, 2005). This period also marked the institutionalization of music education, which laid a solid foundation for this review (Wright et al., 2021). The search was conducted across Web of Science, Scopus, and EBSCO, using the following search string: (“music” AND (“performance” OR “education” OR “instruction” OR “program”) AND (“self-esteem” OR “self-concept”) AND (“student” OR “child” OR “youth”)). Boolean operators and wildcards (e.g., “*”) were applied to maximize coverage. Peer-reviewed studies published from 1970 to 2023 were included, yielding 1,332 records. The search strategy followed the PEO framework and PRISMA guidelines, ensuring comprehensive and reproducible results.

### 2.2 Inclusion/exclusion criteria

To ensure a systematic and rigorous process, the following inclusion criteria were applied: (a) studies published in English; (b) studies focusing on self-esteem within the context of music education; (c) empirical studies published in peer-reviewed journals; (d) studies using standardized self-esteem measurement tools; and (e) studies published between 1970 and 2023. Both quantitative and qualitative studies were included, provided they met these criteria. Studies were excluded if they did not meet the inclusion criteria, including: (a) studies published in languages other than English; (b) studies that did not focus on self-esteem in music education or were unrelated to the research topic; (c) non-peer-reviewed articles, conference proceedings, gray literature, or unpublished studies; (d) studies lacking full-text access or empirical data; and (e) studies published prior to 1970 due to the fragmented nature of early research on self-esteem in music education. The inclusion of empirical studies employing quantitative, qualitative, and mixed-methods approaches was essential, as these methodologies complement each other in enhancing the understanding of self-esteem in music education. Although these approaches are not directly comparable, their integration strengthens the comprehensiveness of the findings, particularly in complex educational contexts (Pearson et al., 2015).

Two reviewers (the first and second authors) independently screened the studies for eligibility based on the inclusion and exclusion criteria to ensure consistency and rigor. Any discrepancies between the reviewers were resolved through discussion until consensus was reached. Only studies published in English were included. This restriction was adopted to ensure accuracy in data interpretation and maintain comparability across studies, thereby avoiding translation-related bias (Balk et al., 2013). Moreover, English-language publications are comprehensively indexed in the international databases employed, ensuring both coverage and feasibility (Neimann Rasmussen and Montgomery, 2018). Given the research team’s linguistic resources and methodological considerations, restricting the review to English-language studies was considered both practical and appropriate.

A total of 1,332 records were retrieved across three databases: Web of Science (*n* = 255), Scopus (*n* = 490), and EBSCOhost (*n* = 587). All records were pooled into a single dataset, followed by duplicate removal and screening using Rayyan, a systematic review screening tool (Ouzzani et al., 2016). This process took approximately four weeks. After duplicate removal and screening, 22 studies were identified as the primary focus of the analysis and marked with an asterisk (*). The exclusion numbers reported at each stage reflect the pooled dataset, in accordance with systematic review standards for transparency and reproducibility (Lefebvre et al., 2019).

### 2.3 Methodological quality evaluation

The articles included in this review were coded using Microsoft Excel, with the coding framework developed based on the research questions and the PEO framework. Detailed information extracted included the publication journal, country, year, research design and methodology, sample size, tools used to measure self-esteem, research findings, and limitations of the studies. To assess the methodological quality of the included studies, we employed an overall Methodological Quality Score (MQS) that rigorously evaluates the validity of the study results (Papaioannou et al., 2016).

**Figure 1:**
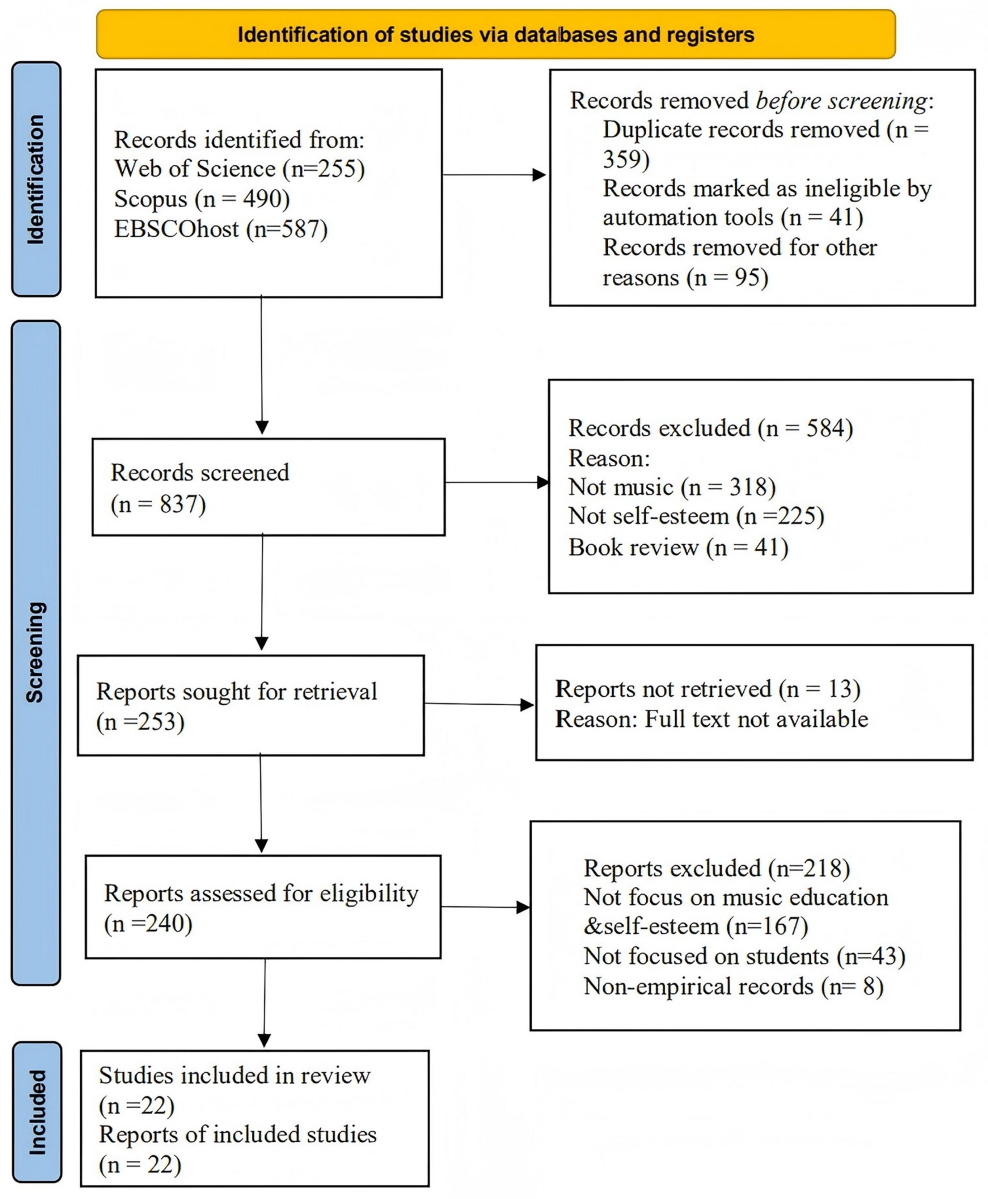
PRISMA flow chart.

We utilized two variations of the quality rating checklist developed by Kmet et al. (2004), which is commonly applied in systematic reviews (Harrison et al., 2021). The checklist comprised 14 quantitative items and 10 qualitative items, each rated on a scale from 0 (not met) to 2 (fully met). These scores were then standardized to a 0–1 scale, with 0.55 as the minimum threshold and 0.75 as a more stringent criterion. For mixed-methods studies, quantitative and qualitative scores were calculated separately. All eligible studies were retained, including those scoring below 0.55, in order to capture the full methodological diversity in the field. Figure 1 presents the PRISMA flow diagram detailing the study selection process (Figure 1).

### 2.4 Reliability and data synthesis

Reliability is a crucial aspect of any systematic review, so we ensured the reliability of both the selection criteria and the coding process. Independent double coding, followed by comparison and random checks, was employed at each stage of the review process, including abstract screening, full-text assessment, quality coding, and data synthesis. Although we did not perform formal inter-rater reliability calculations (e.g., Cohen’s Kappa), repeated discussions and verification minimized subjectivity and ensured consistency in coding decisions (O 'Connor and Joffe, 2020).

To mitigate potential bias, stratified analyses were conducted, and interpretations were made with caution, ensuring that the results reflected methodological variations across studies.

Due to the substantial heterogeneity in study designs, populations, and measurement instruments, a meta-analysis could not be conducted. Therefore, thematic synthesis was employed to develop descriptive and analytical themes across the included studies, a method widely recommended for integrating heterogeneous evidence (Pluye and Hong, 2014). To ensure the integration of qualitative findings, the results of each study were coded and presented in the research outcomes section (Hannes et al., 2018).

## 3 Results

This section provided a comprehensive overview of the outcomes, including data features and themes. By analyzing the themes in each study and examining the content of each topic, one can gain a comprehensive grasp of the themes and identify the recurring patterns throughout the studies to uncover shared themes (Thomas and Harden, 2008). Table 1 provides an overview of the studies, including authors, methodology, participant details, major findings, and other relevant aspects, enabling readers to compare the results and contributions of each study. More detailed information is available in Supplementary material 1, which includes the research objectives, design, methods used (such as surveys and diaries), and other relevant aspects.

**Table 1:** Summary of studies on student self-esteem in music education contexts.

Authors & year	Country	Journal	Methodology	Participants	Duration	Concept & instruments	Major findings	Contributions	Limitations
Anderson and Rickard (2007)	Australia	Australian Journal of Music Education	Quantitative	Seventh-grade students (*N* = 44)	4 weeks	Self-esteem, Coopersmith Inventory	1. No changes in self-esteem or anger. 2. Improved anger control.	No effect on self-esteem or anger.	1. Data loss. 2. Traditional assessments may bias results.
Ark et al. (1980)	United States	Bulletin of the Council for Research in Music Education	Quantitative	Primary school students (*N* = 5,426)	3 months	Self-esteem, Coopersmith Inventory	Self-esteem, social position, and age affect music attitudes.	Self-esteem and social factors influence music attitudes.	Needs further predictive outcome analysis.
Austin (1990)	United States	Contributions to Music Education	Quantitative	Students (grades 5–6) (*N* = 252)	1 academic year	Self-esteem, SEMA scale	Females had higher music self-esteem. 2. Self-esteem related to engagement in music activities.	Music self-esteem linked to participation in music.	1. No clarity on whether self-esteem is a cause or result. 2. Predicted variation modest, with other predictors involved.
Brand (2004)	China	Music Education Research	Quantitative	Full-time undergraduate music education students (*N* = 114)	1 semester	Self-esteem, Self-Description Questionnaire	1. Cultural groupings differ in self-perception. 2. Chinese students have lower self-esteem than Western students.	Cultural variations in self-esteem	None reported
Costa-Giomi (2004)	United States	Psychology of Music	Quantitative	Children (*N* = 117)	3 years	Self-esteem, Coopersmith Inventory	1. Piano improves self-esteem and English performance. 2. No effect on math/ language scores.	Piano lessons boost self-esteem.	None reported
Darrow et al. (2009)	United States	Australian Journal of Music Education	Mixed-methods	High-risk female middle school students (*N* = 24)	several weeks (weekly meetings)	Self-esteem, Culture-Free Self-Esteem Inventory	1. Any musical engagement improves self-esteem. 2. Increased inclination to pursue teaching and assist individuals with disabilities.	Music tutoring improves self-esteem and career orientation	1. Data scoring lacks standardization. 2. Non-randomness. 3. Possible non-blinding.
Draves (2008)	United States	Bulletin of the Council for Research in Music Education	Mixed-methods	Non-music major students (*N* = 20)	1 semester	Music self-esteem, SEMA scale	Positive correlation between self-esteem and musical talent.	Connection between self-esteem and musical talent.	1. Low assessment reliability. 2. Small sample size. 3. Restricted music aptitude in participants.
Egilmez et al. (2019)	Turkey	Universal Journal of Educational Research	Quantitative	Music students (*N* = 66)	1 academic year	Self-esteem, Rosenberg Inventory	1. Weak link between attitude and communication. 2. No link between gender, school, and attitude.	Attitude toward teaching linked to self-esteem.	Alternative methodologies recommended.
Fu and Tu (2023)	China	Frontiers in Psychology	Quantitative	College students (*N* = 362)	8 weeks	Self-esteem, Rosenberg Inventory	1. Music courses improve well-being, but not ethnic identity. 2. Ethnic identity and self-esteem enhance well-being.	Ethnic music courses affect subjective well-being.	1. Geographical constraints. 2. Research needs alternative methodologies.
Hietolahti-Ansten and Kalliopuska (1990)	Finland	Perceptual and Motor Skills	Quantitative	Students (*N* = 55)	6 years	Self-esteem, Battle Inventory	Music students have higher self-esteem.	Engaging in music boosts self-esteem.	None reported
Kruse (2012)	United States	Research Studies in Music Education	Quantitative	Community musicians (*N* = 366)	1 month	Self-esteem, SEMA scale	1. Strong self-worth in music. 2. Disparities in age and interests. 3. No gender-based differences.	Self-worth linked to music involvement.	Variables evaluated via scales, atypical sex ratio.
Lupu (2023)	Romania	Journal Plus Education	Quantitative	Music students (*N* = 150)	3 years	Self-esteem, Rosenberg Inventory	1. Self-esteem linked to academic success. 2. In 2022, relationship with artistic achievement decreased.	Self-esteem improves academic and artistic performance.	Data shows gender disparities, incomplete data.
Michel (1971)	United States	Bulletin of the Council for Research in Music Education	Mixed-methods	Black junior high pupils (*N* = 10)	5 weeks	Self-esteem, Coopersmith Inventory	Music instruction improves self-esteem but limited impact on reading.	Music improves self-esteem in underprivileged students.	Needs clearer transfer scenarios for research.
Michel and Farrell (1973)	United States	Journal of Research in Music Education	Quantitative	Primary school boys (*N* = 14)	10.5 weeks	Self-esteem, Coopersmith Inventory	Music skills may improve self-esteem in disadvantaged students.	Music skills help self-esteem in troubled students.	1. Short intervention duration. 2. Variables should be accounted for.
Otacioglu (2016)	Turkey	Educational Research and Reviews	Quantitative	High school students (*N* = 306)	1 semester	Music self-esteem, SEMA scale	1. No gender difference in music self-esteem. 2. Anxiety varies by gender.	Self-esteem related to music performance.	None reported
Otacioglu (2020)	Turkey	Educational Research and Reviews	Quantitative	College students (*N* = 55)	1 academic year	Self-esteem, Rosenberg Inventory	Self-efficacy correlates with self-esteem.	Self-efficacy linked to self-esteem.	None reported
Randles (2010)	United States	Bulletin of the Council for Research in Music Education	Quantitative	High school band students (*N* = 77)	12 weeks	Music self-concept, SEMA scale	Strong correlation between music self-concept and composition experience.	Musical involvement strengthens self-concept.	1. Correlations alone do not determine causality. 2. More research needed with comparable populations.
Rickard et al. (2013)	Australia	International Journal of Music Education	Quantitative	Students from 2 grades (*N* = 359)	3 years	Self-esteem, Culture-Free Self-Esteem Inventory	1. Music programs prevent declines in self-esteem. 2. Art activities boost self-esteem.	Positive effect of arts on self-esteem.	Non-random assignment, incomplete experimental design.
Şeker (2015)	Turkey	International Online Journal of Educational Sciences	Quantitative	Pre-service music teachers (*N* = 424)	1 semester	Self-esteem, Rosenberg Inventory	1. Weak correlation between self-esteem and attitudes toward music practice. 2. Correlation between self-esteem and academic levels.	Self-esteem linked to music practice.	None reported
Shin (2011)	United States	Contributions to Music Education	Mixed-methods	Middle school students (*N* = 17)	11 weeks	Self-esteem, Self-Description Questionnaire	1. Distinction between school and math self-concept. 2. Program improves self-esteem. 3. Unforeseen impact on musical encounters.	Positive influence of music program on self-esteem.	Project limitations, future research needed.
Sun (2022)	India	Frontiers in Psychology	Quantitative	College students (*N* = 319)	no reported	Self-esteem, Rosenberg Inventory	1. Music improves well-being and academic performance. 2. Self-efficacy and self-esteem are crucial for linking music education to well-being.	Music education enhances well-being and performance.	Alternative sampling and longitudinal methodologies needed.
Zapata and Hargreaves (2018)	Colombia	Psychology of Music	Quantitative	Displaced children (*N* = 52)	18 weeks	Self-esteem, Harter’s Competence Scale	Musical activities improve self-esteem.	Significant positive impact of music.	Unreliable results from one scale dimension.

### 3.1 Journals and countries

Of the 22 studies analyzed, the earliest known study was conducted by Michel in 1971 (Michel, 1971). The study investigated the impact of popular music learning on the self-esteem and academic performance of Black junior high school students from low-income families. It revealed that automated guitar instruction has a beneficial effect on self-esteem in certain instances. However, establishing a clear correlation between self-esteem and academic success has proven challenging. Eighteen studies (81.8%) were published in the last two decades. Over the past five years, specifically from 2019 to 2023, the number of published papers compared to previous years has increased, totaling five articles (22.7%), which indicates an increasing focus on the study of self-esteem in music education. These articles were included in 15 publications and mostly disseminated in academic journals on music education and psychology. Two notable periodicals, namely the “Bulletin of the Council for Research in Music Education” and “Psychology of Music,” are highly regarded for their extensive collection of published studies, which were conducted in eight distinct countries. Most studies were conducted in the United States, accounting for 45.4% of the total (*n* = 10). The results suggest that the knowledge gained from studying self-esteem in the context of music education in the United States could impact perspectives and research directions in wider studies on music education in general. Furthermore, some studies were conducted in Asian countries, including Turkey (18.1%, *n* = 4) and China (9%, *n* = 2). Only one study examined data from several countries and used a cross-country comparative design (Brand, 2004).

### 3.2 Study design, setting, and participants

Among the papers included, 18 followed a quantitative research paradigm, in which 12 employed cross-sectional designs and six conducted intervention trials. These trials consisted of three non-randomized controlled trials, two randomized controlled trials, and one intervention study design. Questionnaires were used for data collection in all quantitative investigations. In addition, the review encompassed four studies that employed mixed-methods, primarily using questionnaires and diaries to gather data. Among them, two utilized cross-sectional designs, whereas the other two were non-randomized controlled trial intervention studies. Pre- and post-intervention testing was done in five of the eight intervention trials (mixed and quantitative methods).

Regarding the number of participants and selection method, 11 studies had small samples with less than 100 participants, and 16 studies employed random selection. Nine studies consisted predominantly of American participants, whereas three studies comprised primarily of African American participants. Eight studies specifically gathered samples from Africa and Asia, with one of these using samples collected from several countries.

### 3.3 Self-esteem assessment instrument

Six of the studies included in the analysis used Rosenberg’s 10-item scale to assess global self-esteem (Rosenberg, 1965). Others used the Coopersmith Self-Esteem Inventory (*n* = 5) (Coopersmith, 1981), the Culture-Free Self-Esteem Inventory (*n* = 3) (Kroner and Sinha, 1989), and a Self-Description Questionnaire (*n* = 2) (Marsh, 1990). One study each utilized the self-esteem subscale of Harter’s Perceived Competence Scale for Children and to assess self-esteem (Harter, 1982). Furthermore, five studies employed the Self-esteem of Music Ability scale to assess a certain type of self-esteem known as musical self-esteem, which is an individual’s personal assessment of their musical abilities, capacity to execute musical tasks, and acquisition of musical abilities (Schmitt, 1979). Assessments of self-esteem primarily concentrate on overall self-esteem and certain facets, such as expression of anger (Anderson and Rickard, 2007) and self-efficacy (Otacioglu, 2020).

Qualitative measurements of self-esteem in mixed-methods studies primarily involve thematic analyses (Draves, 2008). However, only a few studies have assessed and documented the accuracy and consistency of the research findings presented in the publications included. Of all the studies, only 13 included information about the validity or reliability tests conducted on self-esteem measures. Additionally, these studies mainly referenced previously published validation studies on these measures (Sun, 2022).

### 3.4 Assessing the methodological quality of self-esteem research in music education

We evaluated the methodological rigor of the 22 studies included in our analysis. Studies that used both quantitative and qualitative methods were evaluated separately for each component. The findings indicated that the mean score for all quantitative studies was 0.79, whereas for qualitative studies, it was 0.43. Supplementary Table S2 displays the precise evaluation scores for each study. The qualitative components of the four mixed-methods studies had a methodological quality that fell below the minimum worldwide quality criterion of 0.55. Sun (2022)‘s study investigated the impact of music instruction on students’ mental health and academic performance and the role of self-esteem as a mediating factor. It achieved a high ranking in terms of both sample size and data measurement. Meanwhile, Draves (2008) employed surveys and diaries to investigate the connections between music achievement, self-esteem, and aptitude in a songwriting course for undergraduate non-music majors. This study achieved the highest score for qualitative methodological quality among the four mixed-methods studies.

The aims, research questions, and objectives of every quantitative study were sufficiently stated in the evaluation of quantitative studies. Moreover, the assessment of the outcomes and deductions resulted in significant ratings, suggesting that most studies successfully presented both the main and supporting findings, and the conclusions drawn were strongly supported by the data. Previous research has also examined the potential impact of specific individual, contextual, or methodological characteristics on the collected information. For instance, both Randles (2010) and Shin (2011) noted the shortcomings of the studies included in their investigations, which covered a diverse array of topics, and stated that future reviews of the findings should proceed cautiously. In addition, the studies included in our analysis received low scores in terms of adequately detailing the strategies used to select subjects. Most studies provided information solely about the target population without presenting details about the sampling procedure employed (e.g., Costa-Giomi, 2004; Hietolahti-Ansten and Kalliopuska, 1990; Michel, 1971). The values for criteria 6 (“Blinding investigator” = 0.16) and 7 (“Blinding subjects” = 0.16) were excessively low, as most of the studies were conducted as controlled trials. Only one study mentioned the potential for blinding (Darrow et al., 2009), while the remaining studies did not provide any information regarding the possibility of blinding.

The studies included in the analysis had well-defined research questions and objectives, which were determined by evaluating the methodological quality of the mixed-methods qualitative component. These studies also provided a thorough depiction of the surroundings. However, some items had lower scores. Inadequate ratings on Criteria 4, 8, and 10 suggest unclear explanations of data-gathering procedures and data analysis methodologies. For instance, although Michel (1971) and Shin (2011) documented the process of transcribing and coding semi-structured interviews and diaries, they did not disclose the methodology used for coding, nor did they reveal the actual content of the interviews and diaries. In qualitative investigations, researchers do not explicitly evaluate the potential influence of the participants’ personal qualities or the procedures employed on the collected data. Quantitative studies generally performed better in aims, reporting, and conclusions, whereas qualitative studies scored lower, with issues such as limited analytic transparency and insufficient reflexivity constraining interpretability.

### 3.5 Research outcomes

The analysis of the 22 studies yielded several key themes regarding the effects of music education on self-esteem. The findings can be categorized into four main areas: (1) general student populations (*n* = 7), (2) specific student groups (*n* = 5), (3) demographic factors influencing self-esteem outcomes (*n* = 5), and (4) psychological traits and their relationship with music education (*n* = 5).

#### 3.5.1 General student populations (*n* = 7)

Seven studies examined the effects of music programs on the self-esteem of general student populations across different educational stages. Rickard et al. (2013) highlighted the role of school music programs in preventing a decline in self-esteem among elementary school students. Similarly, Hietolahti-Ansten and Kalliopuska (1990) found that participation in music programs had a positive impact on elementary students’ self-esteem. Shin (2011) assessed the effect of a short-term group music program on adolescent self-esteem and anger expression but found no significant impact. At the higher education level, Draves (2008) utilized mixed methods to discover a positive association between songwriting classes and both musical achievement and self-esteem among undergraduate students who were not music majors. Fu and Tu (2023) demonstrated that participation in ethnic music classes improved subjective well-being, self-esteem, and ethnic identity among college students. Two studies explored the influence of instrumental music education programs on student self-esteem. Costa-Giomi (2004), in a controlled experiment, found that children who studied piano for three years significantly improved in academic achievement, self-esteem, and school performance. Randles (2010) reported a correlation between composing music and the self-perception of musical ability among high school instrumentalists, though it was unclear if composition activities directly improved musical self-concepts.

Differences in self-esteem outcomes vary across educational stages, intervention durations, and program types. However, systematic comparisons and analyses of contextual moderators remain scarce, highlighting the need for cautious interpretation and the importance of future studies that rigorously examine these moderating factors.

#### 3.5.2 Specific student groups (*n* = 5)

Five studies focused on the self-esteem outcomes of music education for marginalized groups, such as Black African Americans, at-risk pupils, and students from low-income regions. Michel (1971) investigated the effect of automatic guitar training on the self-esteem of 10 African American junior high school students, finding a positive impact in limited cases. In a follow-up study, Michel and Farrell (1973) discovered that musical talent played a crucial role in enhancing the self-esteem of underprivileged and disturbed children. Darrow et al. (2009) examined the impact of music tutoring on the self-esteem and attitudes of at-risk students, showing a similar increase in self-esteem levels from pre- to post-test, though the results were not statistically significant. Zapata and Hargreaves (2018) provided evidence that engaging in musical activities had a significant positive effect on the self-esteem of displaced children in Colombia. Similarly, Shin (2011) demonstrated the beneficial effects of music programs on the self-esteem of middle school children from low-income neighborhoods through a mixed-methods approach.

The available evidence suggests that music education exerts positive effects on the self-esteem of marginalized groups. However, the small number of studies, limited sample sizes, and heterogeneity in intervention designs and control conditions constrain the robustness and generalizability of these conclusions.

#### 3.5.3 Demographic factors influencing self-esteem outcomes (*n* = 5)

Five studies explored how demographic factors, such as gender, age, and socioeconomic status, influence self-esteem in music education. Ark et al. (1980) examined the relationship between attitudes toward music classes, self-esteem, and demographic features in a sample of 5,426 primary school students. The results indicated that self-esteem varied based on social status, age, and gender. Şeker (2015) observed correlations between the self-esteem levels of pre-service music teachers and their personal characteristics, including gender, age, and their previous educational background. Austin (1990) found that musical self-esteem was a significant predictor of participation in both school and extracurricular music activities, with notable differences in self-esteem between male and female students. Kruse (2012) also investigated self-esteem among adult community musicians, validating that age, gender, and orchestra type were related to self-esteem. However, Otacioglu (2016) found no significant gender differences when exploring the link between musical self-esteem and performance anxiety among music students.

#### 3.5.4 Psychological traits and their relationship with music education (*n* = 5)

Five studies investigated the relationship between self-esteem and psychological traits, such as self-efficacy and self-perception, within the context of music education. Brand (2004) conducted a cross-cultural study in the United States, Australia, and China, finding correlations between self-esteem and cultural self-perception among music education students. Otacioglu (2020) and Randles (2010) both reported a positive relationship between self-esteem and self-efficacy in music majors. Lupu (2023) used a longitudinal design to examine the relationship between self-esteem, academic performance, and artistic achievement among music students before and after the COVID-19 pandemic. Finally, Egilmez et al. (2019) found a strong correlation between self-esteem and communication abilities in pre-service music teachers, though the relationship between self-esteem and attitudes toward music education was less pronounced.

## 4 Discussion

This review sought to deepen understanding of students’ self-esteem in music education by analyzing literature from 1970 to 2023. We evaluated the methodological quality of the studies, focusing on trends, methodologies, and outcomes. Despite a gradual increase in research output, the number of studies remains limited, indicating that this field is still developing. The distribution of studies is heavily skewed toward the United States and Europe, with U. S.-based research representing 45.4% of the total. This imbalance restricts the generalizability of findings to non-Western contexts, emphasizing the need for more culturally diverse studies (Henrich et al., 2010). Additionally, the prevailing self-esteem frameworks are rooted in Western individualist perspectives, which may not adequately address collective identity and social harmony present in non-Western cultures. Future studies should aim to bridge these theoretical gaps and enhance cross-cultural validity.

The sample size in many studies was small, with 11 studies involving fewer than 100 participants. Small samples undermine statistical power (Maxwell, 2004) and limit representativeness, particularly for cross-cultural comparisons. Although small samples can provide exploratory insights, they are insufficient for drawing broad conclusions (Dwan et al., 2008). Future research should prioritize larger, more diverse samples to improve the robustness and external validity of findings.

Most studies employed quantitative methods, with few incorporating qualitative or mixed-methods approaches. While quantitative data are useful, they may not capture the complexity of self-esteem as a construct. We recommend more qualitative and mixed-methods studies to deepen the understanding of self-esteem in music education (Eyisi, 2016; Heyvaert et al., 2013). Such studies would enhance data interpretation by offering both statistical trends and deeper contextual insights. The methodological quality of qualitative studies was often low, mainly due to insufficient transparency in data analysis and lack of researcher reflexivity (Levitt et al., 2017). To strengthen future qualitative research, standardized reporting procedures, such as the COREQ checklist (Tong et al., 2007), inter-rater reliability testing (McHugh, 2012), and triangulation (Fusch and Ness, 2015), should be more rigorously applied.

Meta-analysis was not feasible due to the heterogeneity of study designs, populations, and measurement tools. However, future reviews should consider effect size analysis once a larger body of methodologically comparable studies becomes available. Additionally, more cross-cultural randomized controlled trials (RCTs), longitudinal designs, and intervention studies with rigorous blinding procedures would significantly enhance the robustness and generalizability of findings. Further validation of music self-esteem measures (e.g., SEMA) is also crucial for accurately capturing the mechanisms through which music education influences psychological development.

Several studies relied on single-source surveys, introducing the risk of common source bias (Blair et al., 2013). To mitigate this, future research should employ more rigorous designs, such as field experiments and causal models, and consider longitudinal studies to capture deeper insights (Pearl, 2009). Moreover, intervention studies often lacked post-intervention follow-ups, which are essential for assessing the long-term effects of music education on self-esteem (Hagger and Luszczynska, 2014). Future studies should address this gap and investigate whether improvements in self-esteem are sustained over time.

Although global self-esteem is frequently measured in music education research, the tools used to assess it often lack validity testing, potentially leading to measurement errors (Kimberlin and Winterstein, 2008). The few studies that used the Self-Esteem of Music Ability (SEMA) scale failed to report reliability and validity tests, further reducing the accuracy of results (Abbasi et al., 2024). Future research should prioritize the use of validated tools and thorough reliability testing to enhance data quality (Lewis et al., 2005).

Intervention studies are essential in exploring the impact of music education on self-esteem (Freitas et al., 2022). However, the current review revealed a lack of such studies in the music education context. Given the positive impact of interventions in other fields, future research should investigate specific music education interventions and their effects on self-esteem. Furthermore, to improve the quality of these studies, researchers should ensure proper blinding procedures and employ randomized controlled trials (RCTs) whenever possible (Deeks et al., 2023).

This review highlights the complex relationships between self-esteem development and factors such as demographic characteristics, psychological traits, and student group dynamics in music education. While music instruction consistently boosts self-esteem, especially among marginalized groups, more research is needed to explore these interactions in greater detail. According to self-efficacy theory (Bandura, 1999), key mechanisms like mastery experiences and social persuasion can strengthen students’ self-belief and, consequently, their self-esteem. These effects are particularly significant for disadvantaged students, where musical achievement and positive feedback can foster greater self-efficacy.

Demographic factors, including gender, age, and socio-economic status, also influence how music education impacts self-esteem. For example, gender differences are evident in self-esteem levels, with females often reporting higher self-esteem during adolescence (Bleidorn et al., 2016). These differences may be attributed to greater participation by females in emotionally expressive music activities such as choir, which in turn fosters social belonging and emotional well-being (Milošević, 2024). Age and socio-economic status also shape how music education influences self-esteem, with adolescents and disadvantaged groups benefiting most from music instruction.

Psychological traits, such as self-efficacy, play a key role in shaping self-esteem. Students with higher self-efficacy are more likely to derive benefits from music education, which enhances their mental well-being (Marra, 2019). These findings emphasize the importance of both psychological traits and social engagement in supporting self-esteem growth.

This multi-dimensional interaction between demographic and psychological factors underscores the need for future research to explore how various student characteristics mediate the effects of music education on self-esteem. More studies should focus on non-professional learners and diverse educational settings, including both formal and informal music learning environments (Hallam et al., 2012). By doing so, we can gain a fuller understanding of the ways music education fosters self-esteem across different populations and contexts.

## 5 Limitations and future directions

Although this review provides valuable insights into the impact of music education on self-esteem, several limitations need to be addressed in future research. Methodologically, the review primarily relied on studies published in English, which may introduce language bias. Future research should incorporate multilingual sources and adhere to pre-registration procedures to minimize potential biases.

Substantively, future studies should explore how different types of music, such as traditional and popular music, may foster identity, belonging, and self-worth in various cultural contexts. The evidence in this review indicates that the effects of music education vary according to educational stages, program types, and cultural factors, but further research is needed to understand how these factors differently influence outcomes.

In terms of research methodology, more mixed-methods and qualitative research is needed to capture the complexity of self-esteem. Future studies should focus on larger, more diverse samples, as small sample sizes and single-source surveys have limited the generalizability of many findings. Additionally, more intervention studies, especially those with post-intervention follow-ups, will help assess the long-term effects of music education on self-esteem.

Finally, further validation of self-esteem measurement tools, including the Self-Esteem of Music Ability (SEMA) scale, and the integration of global and multidimensional self-esteem indicators are crucial for improving the accuracy of research on this topic. Longitudinal studies and cross-cultural randomized controlled trials are recommended to better understand the long-term and cross-cultural effects of music education on students’ self-esteem.

## 6 Conclusion

This review systematically synthesized research on students’ self-esteem in music education from 1970 to 2023. The findings demonstrate that music education generally exerts a positive influence on self-esteem, with particularly strong benefits among specific and marginalized groups. However, methodological limitations, restricted cultural coverage, and insufficient theoretical integration remain key challenges.

The review adhered to PRISMA procedures, ensuring transparency and reproducibility in the search and evaluation processes. The evidence indicates that the effects of music education vary across educational stages, program types, and cultural contexts, with stronger outcomes observed in primary school interventions and among marginalized populations. The construct of music self-esteem provides a valuable lens for distinguishing global and domain-specific dimensions and clarifying the mechanisms through which music education promotes self-worth and social connectedness.

In sum, this review updates the research landscape and emphasizes the importance of designing learning environments that intentionally foster self-esteem. Educators and psychologists can adapt elements of music education into targeted interventions, thereby strengthening the evidence base for its role in supporting students’ psychological well-being and social adaptation.

## Data Availability

The original contributions presented in the study are included in the article/Supplementary material, further inquiries can be directed to the corresponding author.
